# A Wearable Multi-Sensor Array Enables the Recording of Heart Sounds in Homecare

**DOI:** 10.3390/s23136241

**Published:** 2023-07-07

**Authors:** Noemi Giordano, Samanta Rosati, Gabriella Balestra, Marco Knaflitz

**Affiliations:** Department of Electronics and Telecommunications and PoliToBIOMedLab, Politecnico di Torino, 10129 Torino, Italy; samanta.rosati@polito.it (S.R.); gabriella.balestra@polito.it (G.B.); marco.knaflitz@polito.it (M.K.)

**Keywords:** wearable sensors, telemedicine, heart failure, electrocardiography, phonocardiography

## Abstract

The home monitoring of patients affected by chronic heart failure (CHF) is of key importance in preventing acute episodes. Nevertheless, no wearable technological solution exists to date. A possibility could be offered by Cardiac Time Intervals extracted from simultaneous recordings of electrocardiographic (ECG) and phonocardiographic (PCG) signals. Nevertheless, the recording of a good-quality PCG signal requires accurate positioning of the stethoscope over the chest, which is unfeasible for a naïve user as the patient. In this work, we propose a solution based on multi-source PCG. We designed a flexible multi-sensor array to enable the recording of heart sounds by inexperienced users. The multi-sensor array is based on a flexible Printed Circuit Board mounting 48 microphones with a high spatial resolution, three electrodes to record an ECG and a Magneto-Inertial Measurement Unit. We validated the usability over a sample population of 42 inexperienced volunteers and found that all subjects could record signals of good to excellent quality. Moreover, we found that the multi-sensor array is suitable for use on a wide population of at-risk patients regardless of their body characteristics. Based on the promising findings of this study, we believe that the described device could enable the home monitoring of CHF patients soon.

## 1. Introduction

Besides the undeniable benefits to healthcare logistics and costs, the spread of telemedicine offers a range of novel diagnostic and therapeutic options. The availability of portable, low-cost, user-friendly sensors enables more pervasive and continuous monitoring of the health status of chronic patients [1]. In this way, a wider population can be targeted, and the patient can be empowered to obtain an accurate follow-up without the need for frequent ambulatory visits [2].

Chronic heart failure (CHF) is a condition that could highly benefit from domiciliary monitoring [3]. In fact, patients affected by CHF, if compliant with the prescribed therapy, can live a relatively normal life until the next episode of decompensation, which goes under the name of acute heart failure (AHF) [4]. AHF is the main contributor to hospital admissions (70% of the total) and mortality [5,6]. It was proved that each new AHF episode further deteriorates the life expectancy of the chronic patient, which decreases from 2.5 years after the first episode to six months after the fourth [5]. 

To enable CHF monitoring in a homecare setting, the main issue resides in finding a biomarker suitable to be monitored in a domicile setting that correlates with the status of decompensation of the heart of the patient. If such a biomarker was available, often, the acute episode could be prevented through a therapy adjustment. Nevertheless, the issue does not have a naïve solution [7,8].

**Figure 1:**
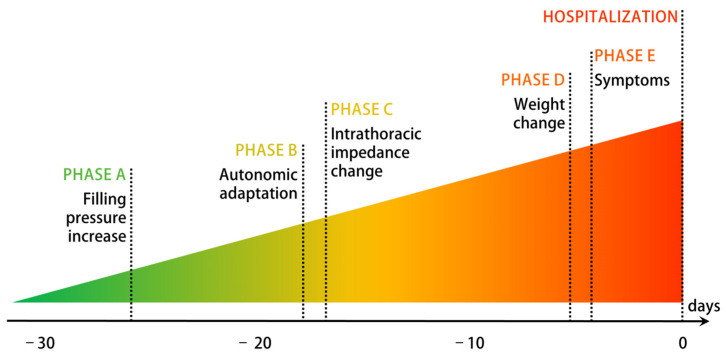
Process of decompensation in CHF patients. Adapted from [9].

Figure 1 proposes an established graphical representation of the decompensation process. As shown, the first changes occur weeks before the AHF episode and the hospital admission and are related to the hemodynamics of the heart [9]. To date, the home monitoring of intracardiac pressures is possible only through invasive hemodynamics sensors. In fact, CardioMEMS™ by Abbott, which is currently the only device trusted by cardiologists, is based on a pressure sensor implanted in the pulmonary artery to monitor the Pulmonary Artery Pressure (PAP) [10,11]. There is a huge interest in finding a noninvasive alternative to CardioMEMS™ with similar predictive value: it would decrease the possible complications related to the implant and reach a much wider at-risk population.

Electro-phonocardiography, i.e., the simultaneous recording of an electrocardiogram (ECG) and a phonocardiogram (PCG), could provide a noninvasive alternative solution. In fact, it was proved that Cardiac Time Intervals (CTIs) extracted from combined ECG-PCG recordings are highly correlated with the Left Ventricular Ejection Fraction and other parameters associated with cardiac functionality in heart failure [12,13,14,15,16,17]. CTIs are temporal hemodynamics parameters that put in relation electrical and mechanical events in the cardiac cycle [18]. Electrical events can be detected in the ECG, whereas some of the relevant mechanical events are detectable in the PCG: the two main heart sounds (S1 and S2) are generated by the closure of respectively the atrioventricular valves (S1) and the semilunar valves (S2). 

Even if electro-phonocardiography has interesting characteristics for the application in home care, such as low cost, portability, and non-invasivity, it lacks, to date, an unavoidable feature: ease of use. The most critical issue resides in the positioning of the microphone sensor. Both with analog and digital stethoscopes, clinical practitioners typically need training to perform reliable auscultation. A reliable positioning is definitely unfeasible for an inexperienced user. It was previously proved that the quality of the signal is affected by the positioning of the stethoscope [19]. Besides, the best auscultation area depends on the application of interest: clinicians typically move the stethoscope to different positions depending on the cardiac valve to be auscultated. The auscultation areas are defined according to the intercostal spaces and other anatomical reference points [20], which are difficult to find for a naïve user, i.e., a person who has no specific clinical or technical knowledge, such as the patient or a caregiver.

The problem of positioning the stethoscope currently limits the applicability of electro-phonocardiography in home care. In this work, we propose a solution to the problem based on multi-source PCG, i.e., the simultaneous recording of multiple PCG signals from different points of the chest. The concept is implemented in a flexible multi-sensor array, which embeds electrodes for ECG recording, 48 microphones at high spatial resolution, and a Magneto-Inertial Measurement Unit (MIMU). 

The goal of this work is to describe the proposed multi-sensor array and demonstrate its usability on a population of inexperienced users with a wide range of body shapes. The usability by people without any clinical or technical skill in the field of auscultation is aimed at showing that the device overcomes the limit of the positioning of the stethoscope in single-source PCG and enables the recording of heart sounds at home. In this sense, the focus of the proposed usability analysis is placed on heart sounds. The possibility of recording heart sounds by inexperienced users, in turn, is expected to enable in the near future the telemonitoring of patients affected by pathologies such as heart failure, which is of great clinical interest.

The proposed approach is innovative because, to the best of our knowledge, it represents the first documented attempt at designing a noninvasive device that can be used directly by the patient or a caregiver and acts at the earliest stage of the decompensation process. In this sense, the relevance of this work resides in demonstrating the main feature of the device that other technological solutions acting at the same stage of the decompensation process are lacking, i.e., its usability by inexperienced users. 

In the following, we will describe the design of the proposed multi-sensor array and the procedure followed for the validation of its usability. We demonstrate that inexperienced users, i.e., volunteers with no technical nor clinical knowledge in the field of auscultation, can successfully use the device to record signals of sufficient quality for the estimation of the time of closure of the cardiac valves. We also demonstrate that the quality of the recordings is partially correlated with the anatomical characteristics of the subject, but a wide chest or a high Body Mass Index do not prevent the user from obtaining a signal of sufficient quality. Overall, we prove that the proposed multi-sensor array enables inexperienced users to record heart sounds in a homecare context, regardless of their body type.

## 2. Design of the Multi-Sensor Array

Little previous experience can be found in the literature about the real-life implementation of ECG and multi-source PCG in a wearable device. The goal of this paragraph is to present the reasoning and the design decisions which led from the idea of the multi-source array to its actual design and realization.

### 2.1. State of the Art

The use of multi-source PCG was explored in the past for different scopes. On one side, it proved valuable for technical tasks such as signal compression [21], noise detection [22], and signal quality improvement [19,23]. On the other side, it has demonstrated superior performances in clinical classification tasks compared to its single-channel counterpart. The multi-channel recording was successfully used to classify subjects with coronary artery disease from normal subjects [24,25,26], to identify and classify cardiac murmurs [27,28], and to estimate the Left Ventricular Ejection Fraction [29]. In the context of CTIs, Paiva et al. employed a two-channel approach to improve the accuracy of their Pre-Ejection Period estimation by selecting the best channel based on a feature related to signal contrast [30]. 

Most of the mentioned studies rely on two to six microphones to be positioned by an expert user considering the traditional anatomical references and are primarily geared towards research rather than real-life domiciliary applications. Only two previous works were found that embed sensors in a device for patient use. Zhang et al. proposed a memory foam pad embedding sixteen microphones to be located on the subject’s chest, primarily optimized for recording lung sounds [31]. Guo et al. designed a vest embedding seventy-two microphones covering the entire thorax to map the propagation of the sound [32]. 

To our best knowledge, no previous examples of a wearable multi-sensor array optimized for the recording of heart sounds using a high number of microphones with high spatial resolution exist in the literature. Additionally, no previous work has explored the use of multi-source PCG with high spatial resolution for CTIs’ estimation.

### 2.2. Conceptualization

The analysis of the state of the art presented in the previous paragraph confirmed that there is a gap in the literature concerning the availability of a device for patient use for the recording of heart sounds. This work aims at filling this gap. As anticipated, the idea of the multi-sensor was triggered by the difficulties in finding a precise location for the microphone sensor. Therefore, the conceptualization began with the definition of the number and position of the microphone sensors. The latter was driven by the definition of the auscultation areas of the four cardiac valves, the result of decades of clinical experience. The traditional auscultation areas are defined as:

- Mitral valve: left hemithorax, fifth intercostal space, along the midclavicular line;
- Tricuspid valve: left hemithorax, fourth intercostal space, along the left sternal border;
- Aortic valve: right hemithorax, second intercostal space, along the right sternal border;
- Pulmonary valve: left hemithorax, second intercostal space, along the left sternal border.

The distribution of the microphones over the chest was defined to cover the above-described areas. The distance between closest neighbors is required to be small enough to ensure that at least some of the microphones fall within the intercostal spaces, even if the positioning of the device does not consider such a constraint. Given the distance between consecutive intercostal spaces, microphones should be located no further than 20 mm from their closest neighbors. In our implementation, we defined a distribution where the concentration of microphones is higher over the traditional auscultation areas in case of good positioning. Nevertheless, microphones are also present elsewhere to deal with the case of bad positioning, covering a 150 mm by 140 mm L-shaped area.

The positioning of the electrodes is not critical in the application of interest because no morphological interpretation of the ECG waveform is required. The ECG is recorded with the scope of identifying the time instant of the ventricular depolarization, which is needed to compute the CTIs. In this scenario, a nonstandard lead can be used, with the scope of having the electrodes positioned close to the microphones without increasing the size of the pad.

Accordingly, the electrodes were positioned as follows:

- First recording electrode: second left intercostal space, along the midclavicular line;
- Second recording electrode: fifth left intercostal space, along the midclavicular line;
- Reference electrode: over the sternum, at the same distance from the two active electrodes.

In our custom-defined nonstandard precordial lead, the two recording electrodes are typically located on opposite sides of the heart to maximize the amplitude of the signal. 

In the end, the MIMU was located over the sternum.

**Figure 2:**
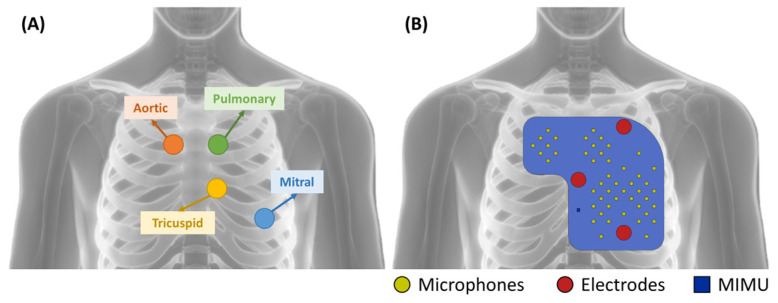
Comparison between (**A**) the traditional auscultation areas and (**B**) the distribution of the sensors in the wearable multi-sensor array.

Figure 2 presents the distribution of the sensors over the device in comparison with the traditional auscultation areas.

The array was implemented in the form of a flexible pad, embedding all the sensors (48 microphones, three electrodes, and one MIMU). In this way, no customization is required. Some degree of flexibility is required to make the pad capable of adapting to the surface of the chest of the subject and grant good contact between the sensors and the skin. Contact is needed for electrodes to allow electrical conduction and for microphones since the refraction coefficient at the skin–air interface is very high, and sound conduction in the air would strongly decrease the intensity of an already weak signal. 

The flexible multi-sensor array can be secured to the thorax by means of an elastic band or, in the future, be integrated into textiles. An elastic band ensures a sufficient fit for the time of the recording, which is expected to be limited to a few minutes a day. No long-term wearing is required.

### 2.3. Sensors and Hardware Design

**Figure 3:**
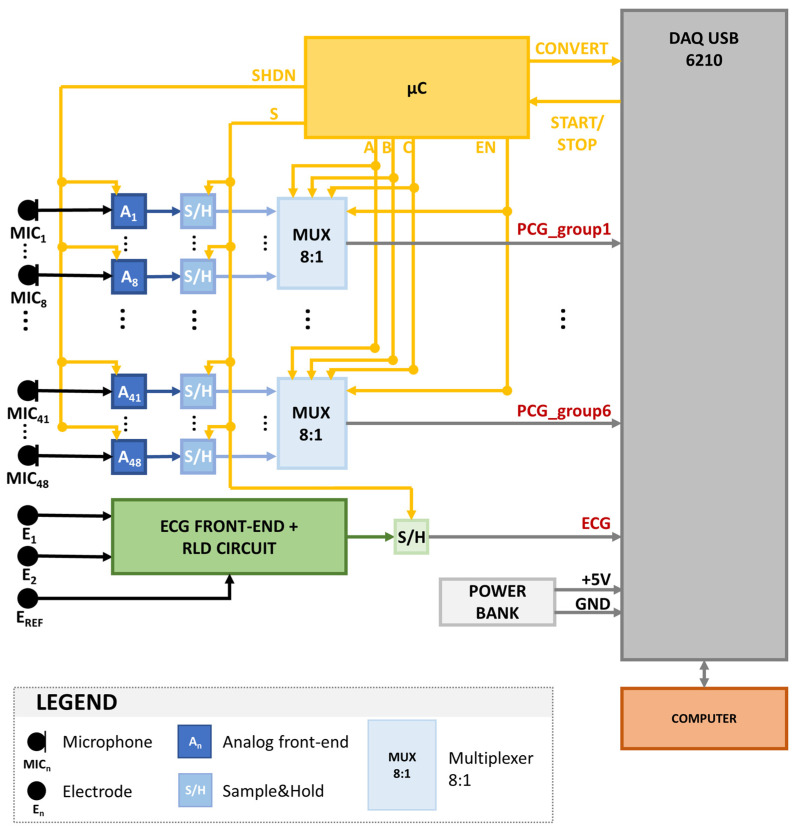
Architecture of the proposed system.

Figure 3 shows a graphical representation of the architecture of the proposed system, designed to validate the functionality and usability of the multi-sensor array.

The heart sounds sensing section grounds on 48 electret condenser microphones. Table 1 presents the main characteristics of the selected microphone sensor (TOM-1537L-HD-R by Pui Audio™, Fairborn, OH, USA).

Among the multiple options on the market, our choice fell on this microphone because it provided the best trade-off between miniaturization (4-mm diameter), which is needed to obtain a grid with high spatial resolution without losing flexibility, and a low high-pass cutoff frequency (20 Hz), which is fundamental for the characteristics of the biomedical signal of interest. In fact, the two main heart sounds span a bandwidth from 20 Hz to 100 Hz. The acoustic signals are pre-amplified on board by 50 dB using the MAX9814 chip by Maxim Integrated™ (San Jose, CA, USA). Moreover, a passive first-order analog high-pass filter is included with a cutoff frequency of 2 Hz to reduce baseline wandering and respiration effects.

The ECG sensing section relies on three custom-made electrodes based on 16-mm-diameter stainless-steel disks. The use of dry electrodes is fundamental to ensure a higher level of ease of use: in this way, no maintenance is required. The electrode conditioning is performed by means of a standard ECG front-end circuit with a differential gain of 380 and a 6 Hz high-pass filtering effect. The common mode reduction is performed by means of the well-known Right Leg Drive (RLD) circuit. 

An LSM9DS1 by ST Microelectronics™ (Geneva, Switzerland) was selected as a MIMU. The latter includes a triaxial accelerometer along with a triaxial gyroscope and a triaxial magnetometer. The recorded signals are output through an SPI. The sensor is embedded in the multi-sensor array for future use (motion detection and posture compensation) but was not used in the first prototype of the complete system.

Both the 48 PCG signals and the ECG signal are sampled by means of as many Sample-and-Hold (S/H) blocks. The same control signal is provided to all S/H, not to introduce any artificial delay in the signals, given that their temporal relationship is the main feature of interest for the forecasted application. All signals are simultaneously sampled with a sampling frequency of 1 kHz to ensure a time resolution of 1 millisecond, suitable for the estimation of the CTIs.

In the first prototype of the device, the signals are multiplexed, converted from analog to digital form, and transferred to a computer by means of a commercial input/output device (DAQ USB 6210 by National Instruments™, Austin, TX, USA). The DAQ card is equipped with eight analog input lines. Therefore the 48 PCG signals were multiplexed using six multiplexer blocks after being sampled. The DAQ USB 6210 converts the signals using a 16-bit Analog to Digital Converter (ADC). All the control signals required to define the timing of the sampling, the multiplexing, and the conversion of the signals are managed by an 8-bit microcontroller (ATmega8 by Microchip Technology™, Chandler, AZ, USA), whose clock is controlled by means of a 16 MHz quartz. 

The NI DAQ USB 6210 card is connected to a computer through the USB port. The recordings are performed by means of a custom, user-friendly Graphical User Interface developed in Python.

### 2.4. Implementation and Mechanical Aspects

The multi-sensor array was implemented in two Printed Circuit Boards (PCBs): a flexible PCB mounting the sensors to be placed over the subject’s chest and a rigid PCB mounting the rest of the electronic components (S/H, multiplexers, microcontroller, quartz). The two PCBs communicate by means of a flat cable.

**Figure 4:**
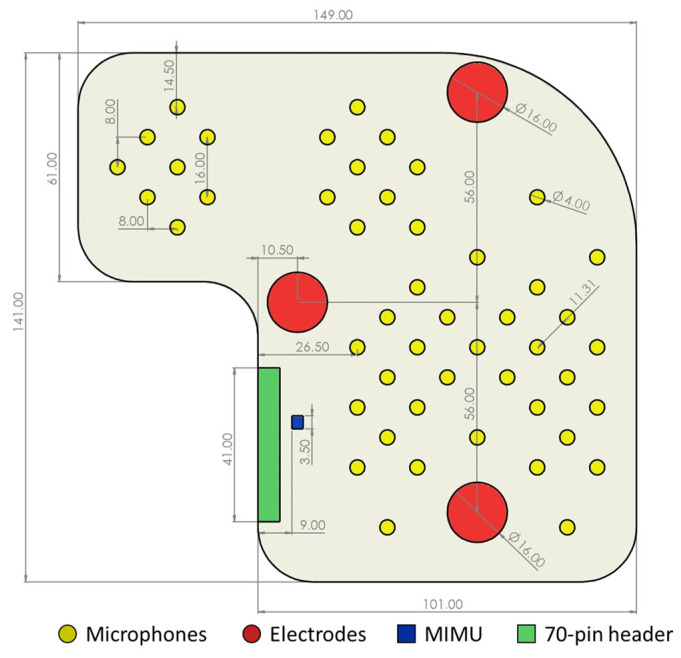
Dimensioned drawing of the multi-sensor array (only relevant dimensions are provided for readability, i.e., dimensions that are either critical for reproducibility or impossible to determine from other dimensions). Dimensions in millimeters.

The flexible PCB was realized using a four-layer Kapton flexible plate. Figure 4 shows the fundamental dimensions, the distribution of the sensors, and their relative distance. The goal was to ensure high flexibility.

**Figure 5:**
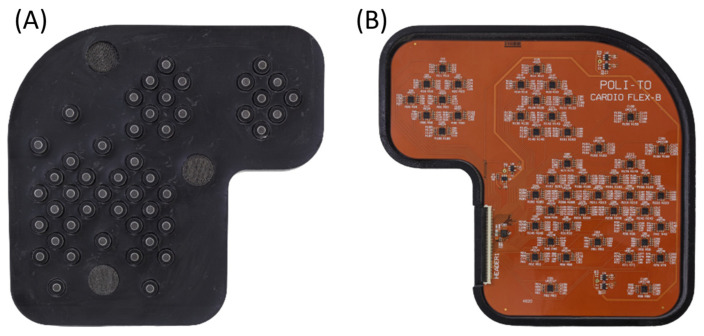
Pictures of the implemented multi-sensor array from the chest side (**A**) and from the top side (**B**).

The PCB was then inserted in a flexible case, which was CAD designed and 3D printed using a biocompatible elastomeric resin (VisiJet^®^ M2E-BK70 by 3D Systems™, Rock Hill, SC, USA). The VisiJet^®^ M2E-BK70 is characterized by a 70 Shore A hardness and by elongation at a break of 42%. Therefore it achieved a good level of flexibility with sufficient resilience to break. Figure 5 presents pictures of respectively the chest side and the top side of the realized multi-sensor array in its 3D-printed case.

The rigid PCB was made in a traditional FR4 4-layer board. The PCB was located in a commercial box by Bopla™ (Bünde, Germany) that could be located next to the subject during the recording. The rationale for separating the remaining electronics from the sensors and the front ends was two-fold. On one side, the minimum possible number of components was located on the multi-sensor array not to unnecessarily increase its rigidity. On the other side, the rigid PCB could be replaced in the future with a standalone microcontroller-based system without the need to modify the multi-sensor array in any way. It should be highlighted that the flexible multi-sensor array is already completely suitable for use in the clinical context and is not limited to a laboratory setting.

## 3. Validation of Usability

The main benefit of the described multi-sensor array with respect to other technological solutions resides in its usability by inexperienced users. Moreover, the multi-sensor array was designed to ensure a good fit with the widest possible pool of at-risk subjects without the need for any customization. The following paragraphs present the procedure that we used to validate the usability of the multi-sensor array.

### 3.1. Experimental Protocol

To validate the usability of the proposed multi-sensor array, couples who were healthy, inexperienced volunteers were enrolled. The experimental protocol was defined with the goal of simulating a real-life homecare scenario, with a caregiver performing a recording on a patient.

In the first phase, in each couple, volunteer A was assigned the role of the patient and volunteer B the role of caregiver. The volunteer caregiver was asked to read a set of simple instructions about how to position the device over the thorax. The volunteer patient was asked to lie on an examination table with a bare thorax and to stay quiet and still throughout the recording. When the volunteer caregiver was ready, he/she was asked to position the array over the chest of the patient, and a 5 min recording was performed. 

In the second phase, the roles were reversed, and a recording of volunteer B (acting the role of the patient) was performed by volunteer A (acting the role of caregiver) with the same modalities. No feedback or any further information was provided to the volunteer caregiver by the investigators to create no bias in his/her way of positioning the device.

The experimental protocol was approved by the Research Ethics Committee of Politecnico di Torino (protocol number 16863/2021).

### 3.2. Signal Processing

For the validation of the usability, the signal processing was limited to two steps: digital filtering and quality assessment. 

Digital filtering was carried out by applying to the raw recordings, channel by channel, a band-pass digital filter between 20 Hz and 100 Hz. The bandwidth was defined according to the bandwidth of the two main heart sounds, S1 and S2, which are required to estimate the CTIs. The filter was designed as an IIR Chebyshev II filter, and zero-phase filtering was implemented to avoid phase distortion. 

The quality assessment was performed by means of the Signal-to-Noise Ratio (SNR) of respectively the first and second heart sound. For each channel and each heartbeat, the SNR was defined as in Equation (1) [33]:

(1) $$SNR=20\;\log_{10}\frac{A_{S}}{4\;\sigma_{N}}$$

where $A_{S}$ is a measure of the signal amplitude (peak-to-peak amplitude of the heart sound of interest), and $4\;\sigma_{N}$ is a measure of the noise amplitude (corresponding to the amplitude of the 95% band of noise). The standard deviation of noise was computed within 70% and 85% of the heart cycle when no heart sound was expected to occur.

### 3.3. Usability by Inexperienced Users

The usability of the device was assessed in terms of the capability of inexperienced users to perform a recording of sufficient quality for the application of interest, i.e., a reliable estimation of the CTIs. On the PCG side, this translates into accurately estimating the time of closure of the cardiac valves from the signals.

The usability by inexperienced users was assessed by evaluating the percentage of recordings performed by the inexperienced enrolled volunteers showing a maximum SNR higher than 13 dB for S1 and higher than 14 dB for S2. In fact, we demonstrated in a previous study that an SNR higher than 13 dB for S1 and higher than 14 dB for S2 ensures an absolute error on the estimate lower than the temporal resolution of the recording system (1 ms) [33]. 

The assessment was carried out, considering the raw signals and the filtered signals, respectively.

### 3.4. Usability on a Wide Range of Body Types

The usability of a wide range of body types was assessed in terms of the possibility of obtaining a good-quality recording regardless of the characteristics of the chest of the patient. This is a measure of the usability of the device and is a critical aspect. In fact, the proposed multi-sensor array is meant to widen the population of at-risk patients under monitoring with respect to its invasive counterpart. Therefore it must ensure a good fit with the widest possible range of body shapes, not limit the target population. 

It can be reasonably hypothesized that the intensity of heart sounds could be decreased due to the presence of a wide chest, a thick layer of interposed fat, or breasts. This is also confirmed by previous studies in the field [34,35]. In this sense, the analysis proposed in this paragraph tackles the problem of the ergonomics of the proposed array. 

Therefore, the correlation between the maximum SNR of respectively S1 and S2 against the BMI, the thoracic circumference, and the gender was assessed. If a correlation is found, a regression line is computed with the scope of defining the limits of applicability of the device. The correlation was computed by means of the Pearson correlation coefficient for continuous variables (BMI, thoracic circumference) and by means of point biserial correlation for categorical variables (gender).

### 3.5. Sample Population

Forty-two volunteers were enrolled in the study. Any clinical or technical knowledge in the field of auscultation was considered as an exclusion criterion to guarantee that all volunteers could be considered inexperienced users. The existence of a present or past cardiac or cardiovascular disease was also considered as an exclusion criterion to guarantee that all volunteers could be considered healthy from a cardiovascular point of view. We highlight that the goal of this work is to demonstrate the usability of the device from a technical point of view: in this sense, the employment of a sample population of healthy subjects instead of CHF patients does not prejudice the validity of the conclusions drawn from this study.

**Figure 6:**
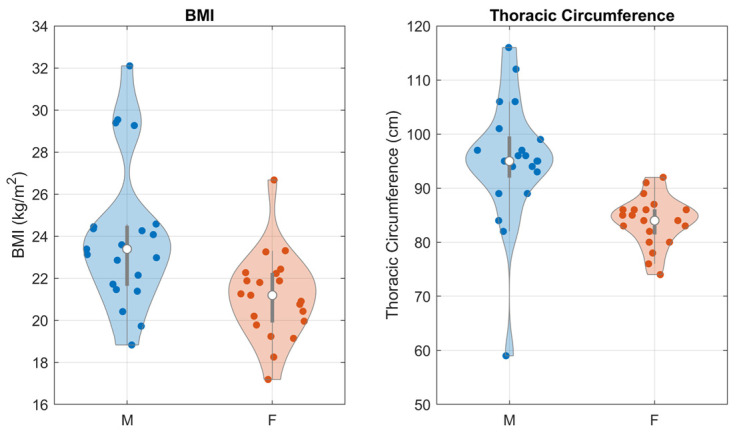
Violin plots of the distributions of respectively BMI and thoracic circumference over the sample population, divided according to biological gender.

The sample population was varied in terms of biological gender (50% females) and body shape, described in terms of Body Mass Index (BMI) and thoracic circumference. Figure 6 presents the distribution of BMI and thoracic circumference in the sample population, as a function of gender. Violin plots were employed to best highlight the shapes of the distributions.

To validate the generalizability of the sample population, the reported anatomical measurements were compared against the corresponding measurements of the general population using the well-established and publicly available DINED 1D anthropometric database by TU Delft [36]. All the measurements were found to be consistent with the database, except for the female thoracic circumference, where the distribution over the sample population was found to have a significantly lower mean and standard deviation. This can be devised as a limitation of this study, but we believe that it does not negatively impact the validity of the results. 

## 4. Results and Discussion

**Figure 7:**
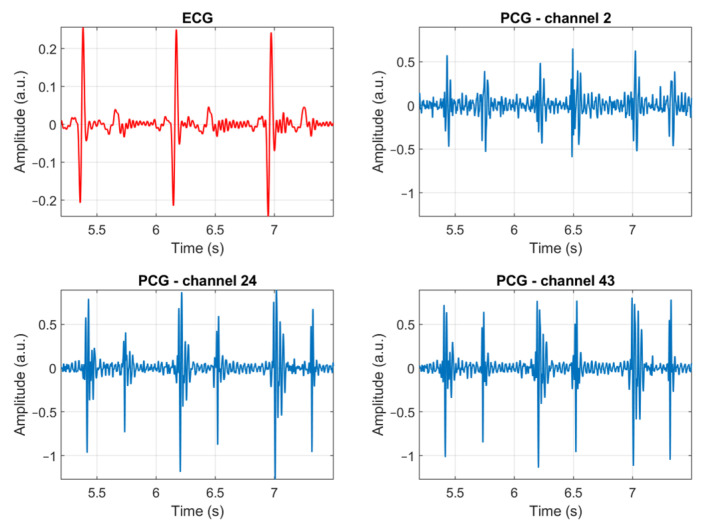
Example of three heartbeats of an ECG signal and three PCG signals, recorded by different channels of the multi-sensor array. No filtering was applied to the signals.

Figure 7 presents three heartbeats of an ECG signal and three PCG signals, recorded by different channels, as an example of the quality of the signals which can be recorded using the proposed multi-sensor array. Three sample recordings are provided in the Supplementary Material (Recordings S1–S3) along with an illustration showing the correspondence between the number of the microphone and its location (Figure S1). The remaining of this Section presents and discusses the results of the validation of the usability of the multi-sensor array on the sample population. The results are meant to provide a quantitative answer to the two-fold question: is the multi-sensor array usable by inexperienced users? Is it suitable for use on a wide range of subjects regardless of their body shape?

### 4.1. Usability by Inexperienced Users

**Figure 8:**
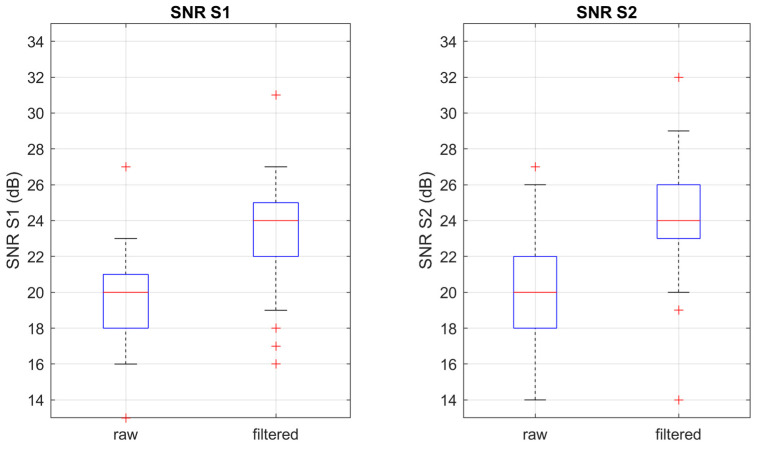
Boxplots of the distributions of the SNR of respectively S1 and S2, before and after digital filtering (20 Hz–100 Hz).

As anticipated, the usability by inexperienced users was assessed by evaluating the percentage of recordings with an above-threshold maximum SNR. Figure 8 shows the distribution of the SNR for, respectively, S1 and S2 before and after a band-pass digital filter between 20 Hz and 100 Hz was applied.

All recordings achieved a maximum SNR above the threshold even before any digital filtering was applied. We can conclude that at least a good-quality signal could be recorded by 100% of the inexperienced users in the sample population. Moreover, the median maximum SNR in the filtered signal was found to be as high as 24 dB for both S1 and S2, which can be considered excellent quality. Therefore, the multi-sensor array achieves its primary goal, i.e., obtaining a PCG signal of good to excellent quality even if the device is positioned by a user with no clinical or technical skills. 

As a second-level analysis, we evaluated how many channels presented an above-threshold SNR. This information is interesting because a high number of good-quality channels allows for a selection of the best channel based on the valve of interest. It was found that more than half of the channels presented an above-threshold SNR. In particular, the average percentage of channels presenting an above-threshold SNR was found to be as high as 55% for S1 and 64% for S2 over the sample population. Therefore, the multi-sensor array not only enables inexperienced users to obtain a good-quality recording, but it may also interestingly allow for differentiating among different auscultation areas. 

**Figure 9:**
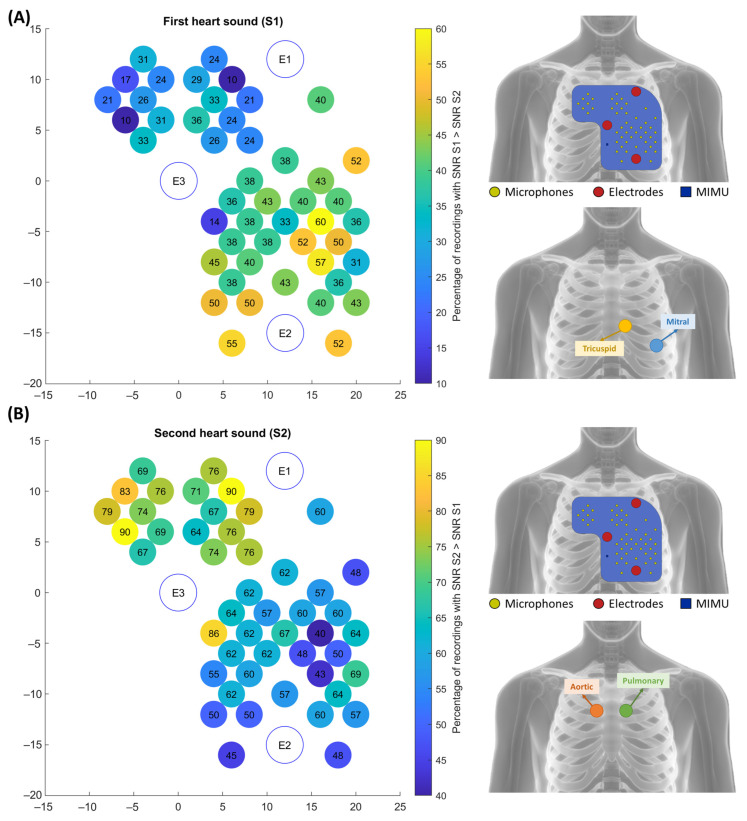
Maps showing the percentage of recordings with respectively a higher SNR in S1 (panel **A**) and a higher SNR in S2 (panel **B**). Each circle represents a microphone, and the color of the circle represents the percentage of recordings according to the color bars. A comparison between the positioning on the chest of the best channels against the positioning of the traditional auscultation areas of the valves generating the corresponding heart sound is proposed.

To confirm this point, the SNR values of S1 and S2 were compared channel by channel. Figure 9 shows the percentage of recordings with a higher SNR in respectively S1 and S2. The S1 map shows that the best quality signals when considering S1 are located in the lower part of the left hemithorax. This is coherent with what is expected from the clinical practice since the mitral and tricuspid auscultation points are located in that area. Similarly, the S2 maps show that the best quality signals, when considering S2, are located in the upper part of the array, all across the sternum. In addition, in this case, the experimental findings are coherent with the theoretical aortic and pulmonary auscultation areas. 

To resume, we can conclude that not only all 42 inexperienced users were capable of obtaining recordings with good to excellent quality, but the distribution of the SNR over the chest was also coherent with the expected auscultation areas. In this sense, the multi-sensor array could provide the user with even more information than a traditional single-source stethoscope and allow for differentiating the different auscultation areas depending on the requirements of the application of interest (e.g., depending on the CTI to be estimated).

### 4.2. Usability on a Wide Range of Body Types

**Figure 10:**
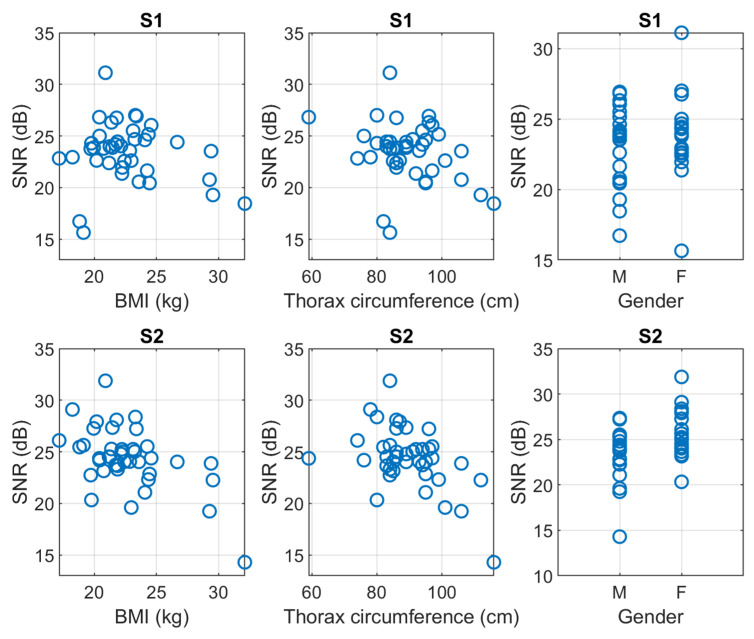
Scatter plots of the maximum SNR of respectively S1 and S2, in function of, respectively, the BMI, the thoracic circumference, and the biological sex.

Figure 10 plots the obtained maximum SNR for S1 and S2, respectively, in the function of the three parameters that we used to give a quantitative interpretation to the body type, i.e., the BMI, the thoracic circumference, and the gender.

The correlation coefficients between the SNR of S1 and S2 and the three parameters of the body type are shown in Table 2. The SNR was computed on filtered signals.

To correctly interpret the results, it should be highlighted that the BMI and the thoracic circumference were found to be strongly correlated, with a correlation coefficient of 0.82. Nevertheless, this was not obvious in the first place because BMI represents a global anatomical feature, whereas the thoracic circumference is a local one. 

Results show that the quality of S1 depends on none of the considered anatomical features, whereas the quality of S2 weakly depends on gender and moderately depends on BMI and thoracic circumference. 

We can state that the characteristics of the designed multi-sensor array ensure the possibility of recording good-quality ECG-PCG signals on the large majority of the population regardless of their body type. This confirms that the device’s ergonomics make it usable on a wide population and does not limit the applicability to a specific body shape.

## 5. Conclusions

In this work, we proposed a novel multi-sensor array devoted to enabling the simultaneous recording of ECG and PCG signals in a homecare setting by the patient himself or by an inexperienced caregiver. A ready application of the array is the domiciliary telemonitoring of the status of compensation of patients affected by CHF: we know from the literature [12,13,14,15,16,17] that changes in the CTIs, extracted from the signals recorded through the multi-sensor array, reflect changes in the intracardiac pressures and thus of the cardiac functionality of the patient.

It should be highlighted that other technological solutions were explored to deal with the problem of the noninvasive monitoring of CHF patients. Nevertheless, they either act at a later stage of the decompensation process (such as the phase of systemic and pulmonary congestion), or they are ground on similar assumptions than we do but are devised for ambulatory use because they cannot be used by the patient or a caregiver.

The main reason for proposing the multi-sensor array, where the innovativeness of our project resides, is its theoretical usability by inexperienced users, which we aimed to demonstrate in this work. We validated this point by having 42 inexperienced users use the device on each other in a simulation of real-life use. All subjects could obtain heart sounds of good to excellent quality. Moreover, the array allowed for differentiation among different auscultation areas, opening novel possibilities in terms of signal processing.

In the end, the multi-sensor array proved capable of functioning on a wide range of different body types. In fact, besides the signal quality being partially correlated with the BMI and the thoracic circumference, as expected, none of these anatomical features affected the possibility of obtaining a good-quality signal in all the tested subjects. This is a relevant characteristic to ensure the maximum coverage of the at-risk population. 

The use of ECG and PCG signals may have some limitations that we dealt with in our project. Concerning the ECG signals, the main limitation resides in the use of a nonstandard single lead: for this reason, the morphological interpretability of the recorded ECG is affected, and the latter cannot be considered suitable for diagnosis. Nevertheless, the latter limitation does not affect the use of our recorded ECG for the estimate of the CTIs, since, in this case, the time instant corresponding to the depolarization of the ventricles is required, and the latter is not affected by the use of a nonstandard lead. Concerning the PCG, the main limitation resides in the dependence of the quality and significance of the signal on the positioning of the sensor over the chest. The use of multi-source PCG was explored to overcome this limitation and represents the main takeaway of this work.

The main limitation of this study is testing on a rather small sample population, which partially corresponds to the general population in terms of anthropometric measurements. Future developments include the testing on subjects affected by a range of cardiovascular conditions as well as the validation of its final real-life application. We can forecast that, in the near future, the described multi-sensor array could be used for the telemonitoring of CHF patients in a homecare context.

## 6. Patents

The multi-sensor array described in this work is the object of the patent “Multi-sensor wearable active array for the prevention of heart failure”—Owner: Politecnico di Torino, Inventors: Marco Knaflitz, Noemi Giordano, Priority number: 102020000014428, Priority date: 17 June 2020. https://www.knowledge-share.eu/en/patent/multi-sensor-wearable-active-array-for-the-prevention-of-heart-failure/ (accessed on 15 June 2023)

## Figures and Tables

**Table 1:** Characteristics of the microphone sensors ^1^.

Parameter	Value
Diameter	4 mm
Sensitivity	−37 dB ± 3 dB
Signal-to-Noise Ratio	68 dB
Frequency Range	20 Hz–20 kHz
Output Impedance	2.2 kΩ
Directivity	Omnidirectional

^1^ From the datasheet of the component, the TOM-1537L-HD-R by Pui Audio™.

**Table 2:** Correlation between the SNR of the heart sounds and the quantitative parameters of the body shape ^1^.

Anatomical Parameter	Correlation with SNR S1	Correlation with SNR S2
Gender	0.00 (−0.30–0.31)	* 0.35 (0.06–0.60)
BMI	−0.03 (−0.33–0.28)	* −0.53 (−0.72–−0.27)
Thoracic circumference	−0.16 (−0.44–0.15)	* −0.48 (−0.68–−0.21)

^1^ Correlation coefficients computed as Pearson correlation coefficients for continuous variables (BMI and thorax circumference), point biserial correlation coefficients for categorical variables (gender). The * symbol shows what correlations are statistically significant with α = 0.05. The interval in brackets represents the 95% confidence interval of each correlation coefficient.

## Data Availability

The data presented in this study are available on request from the corresponding author.

## Supplementary Materials

The following supporting information can be downloaded at: https://www.mdpi.com/article/10.3390/s23136241/s1, Recording S1: sample recording number 1; Recording S2: sample recording number 2; Recording S3: sample recording number 3; Figure S1: correspondence between the number of the microphone and its location. Recordings are mat structures containing the following fields: rec.ecg (column array containing the raw ECG signal), rec.pcgs (matrix containing the 48 raw PCG signals on the columns), rec.time (column array containing the time instants), rec.fs (value of the sampling frequency in Hz).
