# Supplementary figures and images for: A Wearable Multi-Sensor Array Enables the Recording of Heart Sounds in Homecare
Source: Sensors (Basel). 2023 Jul 7;23(13):6241. doi: 10.3390/s23136241 (PMC10346306; doi:10.3390/s23136241)

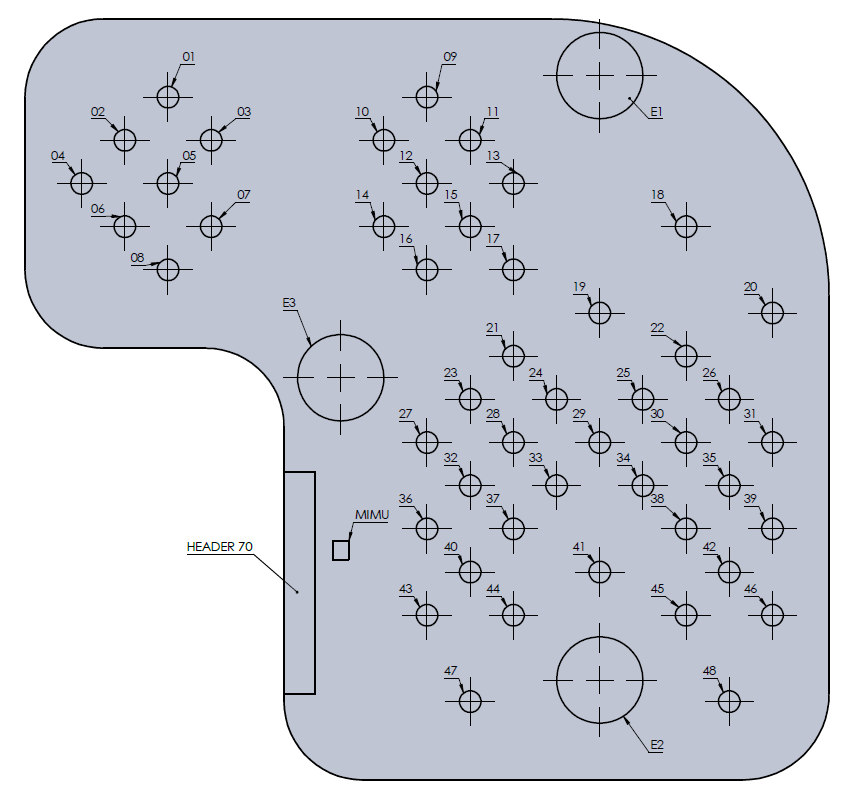

Supplement: Supplementary file 1 [file sensors-23-06241-s001.zip › Figure S1_sensor_locations.png]
